# Vortex Flow on the Surface Generated by the Onset of a Buoyancy-Induced Non-Boussinesq Convection in the Bulk of a Normal Liquid Helium

**DOI:** 10.3390/ma14247514

**Published:** 2021-12-08

**Authors:** Alexander Pelmenev, Alexander Levchenko, Leonid Mezhov-Deglin

**Affiliations:** 1 Institute of Solid State Physics RAS, 142432 Chernogolovka, Russia; levch@issp.ac.ru (A.L.); mezhov@issp.ac.ru (L.M.-D.); 2 Chernogolovka Branch of Federal Research Centre for Chemical Physics RAS, 142432 Chernogolovka, Russia; 3 L.D. Landau Institute of Theoretical Physics RAS, 142432 Chernogolovka, Russia

**Keywords:** convection, heat and mass transfer, free surface patterns, vortex flow

## Abstract

The onset of the Rayleigh–Benard convection (RBC) in a heated from above normal He-I layer in a cylindrical vessel in the temperature range T_λ_ < T ≤ T_m_ (RBC in non-Oberbeck–Boussinesq approximation) is attended by the emergence of a number of vortices on the free liquid surface. Here, T_λ_ = 2.1768 K is the temperature of the superfluid He-II–normal He-I phase transition, and the liquid density passes through a well-pronounced maximum at T_m_ ≈ T_λ_ + 6 mK. The inner vessel diameter was D = 12.4 cm, and the helium layer thickness was *h* ≈ 2.5 cm. The mutual interaction of the vortices between each other and their interaction with turbulent structures appeared in the layer volume during the RBC development gave rise to the formation of a vortex dipole (two large-scale vortices) on the surface. Characteristic sizes of the vortices were limited by the vessel diameter. The formation of large-scale vortices with characteristic sizes twice larger than the layer thickness can be attributed to the arising an inverse vortex cascade on the two-dimensional layer surface. Moreover, when the layer temperature exceeds T_m_, convective flows in the volume decay. In the absence of the energy pumping from the bulk, the total energy of the vortex system on the surface decreases with time according to a power law.

## 1. Introduction

The convective heat transfer in fluids and dense gases plays a role in nature (convection in planets’ atmospheres, in the bulk of oceans, and eruptions of volcanoes) and engineering. For this reason, the thermogravitational RBC in various media is being actively studied in numerous works [1,2]. The most recent developments about the transition to turbulence in convection can be found in the reviews [3,4,5,6].

Liquid helium is one of the most convenient model objects for the laboratory studies of the Rayleigh–Benard convection [7], as well as for the investigation of wave and vortex turbulent phenomena in the bulk and on the surface of liquids. 

Most previous measurements were taken in a confined geometry. A liquid layer was placed between two solid plates made of high thermal conductivity materials and under the so-called Oberbeck–Bussinesq approximation. The thermal expansion coefficient is positive β = −(1/ρ) (∂ρ/∂T) > 0, the temperature gradient ∂T/∂z is positive and is directed opposite to a gravitational field, and other thermodynamic coefficients almost do not depend on temperature. 

**Figure 1:**
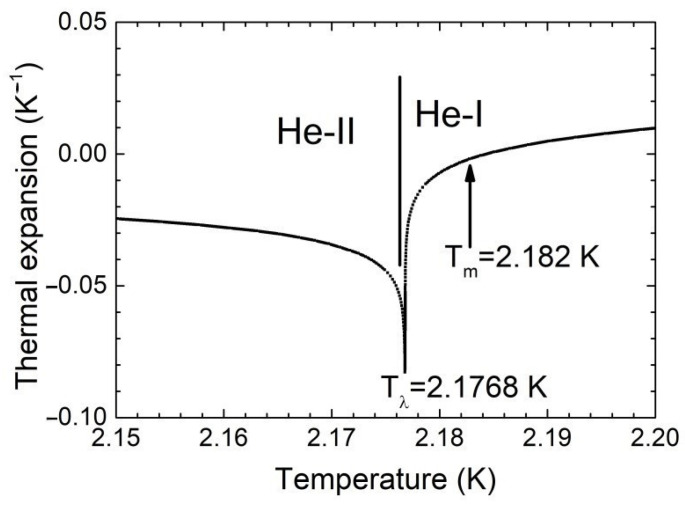
Temperature dependence of the thermal expansion coefficient of liquid ^4^He near T_λ_.

However, in the series of our recent experiments [8,9] we have studied pattern phenomena on the free normal helium surface in the process of the RBC onset under the strong non-Oberbeck-Boussinesq conditions: the liquid helium layer was heated from above in a narrow temperature range T_λ_ < T ≤ (T_λ_ + 6 mK) where the liquid helium density passes through a maximum [10] (see Figure 1). The thermal expansion coefficient of liquid ^4^He is negative β < 0 in the range of 1.4 K < T < T_m_, and it passes through zero and becomes positive β > 0 at T > T_m_, as in most convenient liquids

**Figure 2:**
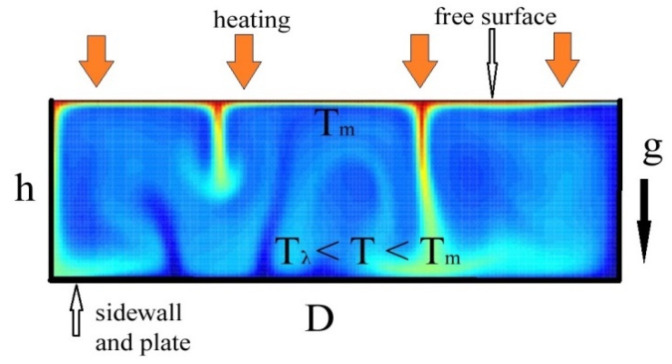
Illustration of the onset of the natural thermal RBC in the bulk of the normal helium layer heated from above in a narrow temperature range near T_λ_ in gravity field g.

In the volume of the He-I layer heated from above in the temperature range ΔT = T_m_ − T_λ_ ≈ 6 mK the RBC is developed due to a buoyancy-driven flow. The fluid layer in the wide cylindrical vessel is set into motion by natural convection as shown in Figure 2. The temperature of the liquid near the vessel bottom T_b_ is less than that on the free liquid surface T_s_ ≤ T_m_. Blue hues demonstrate cold areas while the red hues are hot. A cold, less-dense lower boundary layer sends plumes of a cold liquid upwards, and likewise, a warm liquid moves from the top downwards.

In the experiments discussed below the inner diameter of the vessel was D = 12.4 cm and thickness of the liquid layer was h ≈ 2.5 cm, thus the aspect ratio was G = D/*h* ≈ 5.

Using the known values of the liquid helium [10], the density ρ = 0.14 g/cm^3^, the liquid viscosity ν ≈ 1.4 × 10^−4^ cm^2^/s, and the thermal diffusivity *χ =* ϰ*/ρC_p_* ≈ 2.5 × 10^−4^ cm^2^/s (here ϰ is the thermal conductivity and *C_p_* is the heat capacity), the thermal expansion coefficient is negative and close to β ≈ −0.018 K^−1^ at T ≈ 2.178 K, and assuming that the difference in temperatures is ΔT ≈ 3 mK, one can estimate the dimensionless parameters the Prandtl number P = ν/*χ* ≈ 0.56, and the Rayleigh number Ra = *g* ∆Th^3^ β/(νχ) ≥ 10^7^.

The estimated Ra value is four orders of magnitude higher than the critical Rah number Ra_c_ ≈ 1.1 × 10^3^ found for a case of a free upper liquid surface. In other words, turbulent vertical convection should be established with time in the He-I layer volume [1,2,3,4,5,6].

## 2. Materials and Methods

### 2.1. Materials and Experimental Method

**Figure 3:**
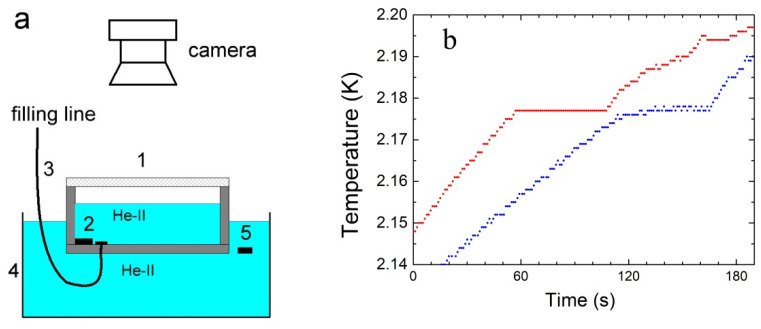
(**a**) Scheme of the experimental setup: 1-working cylindrical vessel with a Plexiglas window; 2, 5-thermometers; 3-filling line; 4-auxiliary bath. (**b**) The upper curve demonstrates the time dependence of the liquid temperature near the bottom of the vessel T_b_ (red line), and the lower curve is the liquid temperature in the auxiliary bath T_a_ (blue line) after switching off the external mechanical pump. The He-II to He-I phase transition inside the vessel occurs at a time of ~55 s.

The inset to a metallic cryostat specially designed for studying nonlinear phenomena on the free liquid helium surface was earlier described in [8,9]. The schematic of the measurements is presented in Figure 3a. The characteristic dimensions of the duralumin cylindrical vessel 1 are: the inner diameter D = 12.4 cm, the wall thickness l = 0.2 cm, and the height of 4 cm. The vessel was hermetically sealed with the transparent Plexiglass window (with the thickness of ~0.8 cm). The stainless steel capillary 3 was used to fill vessel 1 with high pure gaseous ^4^He. Thermometers 2 and 5 were used to monitor the liquid helium temperature at the bottom of vessel 1 and in the auxiliary bath 4, respectively.

Vessel 1 was filled with liquid helium by the condensation of gaseous helium through capillary 3 when the temperature of liquid helium in the cryostat bath was T = 4.2 K. Then, the liquid temperature in the cryostat and auxiliary bath 4 decreased down to T < T_λ_ by pumping the helium vapors with an external mechanical pump.

On achieving the thermal equilibrium in the cryostat bulk the pump was turned off, and the temperature of superfluid He-II in the cryostat began to increase smoothly. The radiative heat flux emitted by the warm upper cap of the cryostat was adsorbs almost entirely by the Plexiglas window 1. Next, the heat adsorbed by the window propagated over the helium vapor and the vessel sidewalls toward the liquid helium surface inside the vessel 1 (Figure 2).

The plateau on red line in Figure 3b (a time range of ~60–110 s) corresponds to the development of the turbulent convective flows in the normal helium layer heated from above in the temperature range of T_λ_ < T ≤ T_m_. With raising the liquid temperature near the vessel bottom above T_m_ the liquid density near the bottom exceeds that on the layer surface. Moreover, under these conditions (β > 0), convective flows in the bulk should decay quickly. Because of this, the effective thermal conductivity of the He-I layer decreases, and well pronounced kinks occur on red line (time of ~110 s). Analogous dependence on time of the helium layer temperature in a glass dewar during the RBC onset was observed by Peshkov [11].

It is seen in Figure 3b that at the beginning of measurements the temperature of He-II in the vessel T_b_ (red line) is noticeably higher than that of the liquid in the auxiliary bath T_a_ (blue line): superfluid helium in the vessel 1 is overheated in comparison with the liquid in the auxiliary bath, and ∆T = T_b_ –T_a_ ≈ 15 mK. It can be used to estimate by an order of magnitude the heat flux density Q_c_ propagating through the He-II layer in vessel 1 to auxiliary bath 4. Using the literature values of the Kapitza resistance at the metal-liquid helium boundary R_k_ ≈ 20/T^3^ (Kcm^2^/W) [12], the thermal conductivity of duralumin walls ϰ_w_ ≈ 2 × 10^−2^ W/cm K at T ≈ 2 K [13] and the vessel wall thickness l = 0.2 cm one can write ∆T = 2 ∗ (Q_c_ ∗ R_k_) + Q_c_ ∗ l/ϰ_w_ = Q_c_ ∗ (2 ∗ R_k_ + l/ϰ_w_) ≈ 15 ∗ Q_c_, thus, by an order of magnitude Q_c_ ≈ ∆T/15 ≤ 1× 10^−3^ W/cm^2^. Assuming that the density of the heat flux passing through the free surface of the helium layer to the vessel bottom Q_c_ does not change significantly on heating the liquid above T_λ_, the temperature difference on the layer surface and near the bottom is ΔT_c_ ≈ 3 ÷ 6 mK, and the thermal conductivity of He-I near the bottom is ϰ_h_ ≈ 4 mW/cm K, one could estimate the Nusselt number. The ratio of the convective Q_c_ to the conductive Q_d_ = ϰ (ΔT_c_/h) heat flux transfer across a boundary is N_u_ = Q_c_/Q_d_ = Q_c_ h/ϰ_h_ΔT_c_ ≤ 10^2^. In principle, this estimate agrees with the estimates of the Nusselt number from the relations between the Rayleigh and Nusselt numbers given in the literature for large R_a_ [3,4]: at R_a_ ≈ 10^6^–10^8^ the ratio N_u_/(R_a_ )^1/3^ ≈ 0.08, or at R_a_ ≈ 10^7^ the Nusselt number should be N_u_ ≈ 20.

It is known that the N_u_ value between 1 and 10 corresponds to of a slug flow or a laminar flow, and the larger Nu numbers correspond to a turbulent flow (the N_u_ values fall between 100–1000 [1,2,14]).

### 2.2. Image Acquisition and PIV Measurements

To study any motion on the free surface of liquid helium, we used light 50 μm glass microspheres. It was observed that the glass spheres had combined into small aggregates (tracers) with an average diameter of 0.1–0.3 cm under the liquid surface [15].

To record the motion of tracers on the helium surface we installed the video camera above the transparent top of the cryostat. The frame rate of the video was equal to 24 fps. In order to process the video records and to identify the trajectories of the traces on the liquid surface, we have used the well-known PIVLab software [8,9,15,16]. The images processing made it possible to estimate the velocities of the tracer movement on the liquid surface V_x_ and V_y_, the tracers trajectories, and then to calculate the vertical vorticity Ω on the helium surface, the energy distribution E(x, y) over the surface, and total kinetic energy of the vortex motion E on the surface.

The vertical vorticity on the liquid surface Ω was determined as (Equation (1)):

Ω = dV_x_/dy − dV_y_/dx (1)

The energy distribution over the surface was calculated with Equation (2):

E(x,y) = ½ ∗ ρ ∗ (V_x_^2^ + V_y_^2^ ) (2)

The energy distribution E(k) over the wave vector in the k-space was estimated by averaging over the ring (Equation (3)):

(k) = 1/(2S∆k)ʃd^2^q/(2π)^2^ [|V_k_|^2^] (3)

The integration was performed over the ring k < q < k + ∆k, and the resulting value was normalized to the liquid surface area S. Here, V_k_ is the Fourier component of the tracer velocity. The brackets ‘[]’ denote averaging over the frames taken at different times.

## 3. Results and discussion

**Figure 4:**
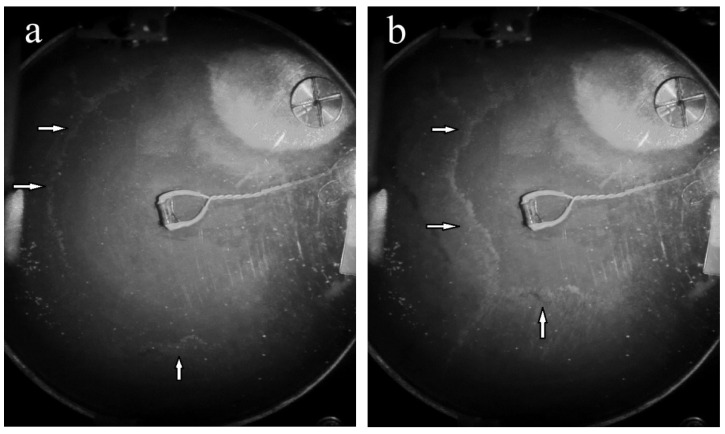
The tracers front on the liquid surface is moving from the sidewalls to the vessel center at times of ~2 s (**a**) and ~3 s (**b**) after the moment of the superfluid He-II—normal helium He-I phase transition. The tracer front is marked by white arrows.

The direct observations showed that at the first seconds after the passage of the temperature through the critical temperature T_λ_, the video camera had recorded the appearance of an intense flow on the surface directed from the sidewalls to the vessel centre (Figure 4). The centripetal velocity of the tracer front moving from the vessel walls was V~2 cm/s. The Reynolds number of the tracer motion reaches Re~(VL)/ν∼10^4^, which indicates the possibility of vortex flow appearance on the liquid surface.

**Figure 5:**
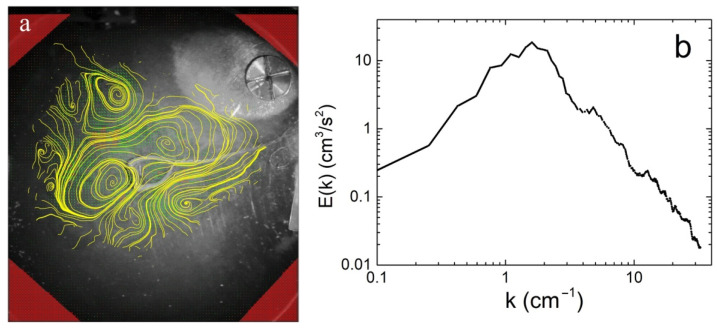
(**a**) Streamlines on the He-I surface ~40 s after the He-II–He-I phase transition. (**b**) Energy distribution of the vortex system E(k) in the k space at this time.

The tracer front collapsed near the vessel center, and then a number of vortices of different sizes appeared on the surface. The vortices were rotated in opposite directions. Figure 5a shows vortices on the liquid surface ~40 s after the He-II–He-I transition. The energy distribution of the vortex system E(k) in the k space at this time is illustrated Figure 5b.

**Figure 6:**
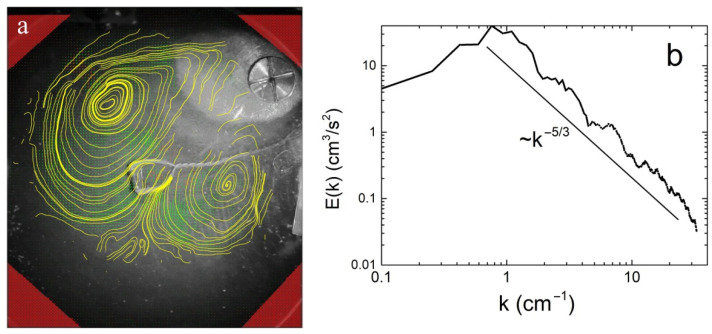
(**a**) Current lines on the He-I surface ~80 s after the He-II–He-I transition. (**b**) Energy distribution E(k) of the vortex system in the k space at this time. The straight line corresponds to the dependence E(k)~k^−5/3^.

It is clearly seen that several vortices rotating in opposite directions are excited on the liquid surface at this time. The maximum energy in the E(k) spectrum is at the wave vector k of ~1.6 cm^−1^ (Figure 5b). The estimated from the Figure 5 value of the vorticity Ω in a single vortex reaches about ~3 s^−1^. Using the calculated from experiment values of the vortex dimensions L~1.5 cm and the viscosity of liquid helium, the Reynolds number can be estimated: Re~(ΩL^2^/ν)~4 × 10^4^. A high Re value indicates that the vortex motion on the liquid surface excited by convective motion in the layer is highly nonlinear, that points to a strong interaction between the vortices. Moreover, due to this by 80 s the distributions of vortices along the layer surface has changed significantly, and two large vortices rotating in opposite directions have been formed. Vortex dipole occupies almost the entire surface of the liquid in the vessel. The corresponding streamlines and the energy distribution E(k) of the vortex motion on the surface at this time are shown in Figure 6. The energy of the vortex motion on the surface reaches its maximum value.

The appearance on the He-I surface of two large-scale vortices with characteristic dimensions exceeding the thickness of the liquid layer by more than two times indicated the formation of an inverse vortex energy cascade on the surface of the quasi-two-dimensional liquid layer. This is also evidenced by the observed power-law dependence of the energy distribution of the vortex system over the wave vectors E(k)~k^−5/3^ (Figure 6), which is characteristic of the appearance of an inverse energy cascade in a two-dimensional layer. The position of the energy maximum in the spectrum E(k) shifts towards small k (k~0.76 cm^−1^), which is also pointed out to the formation of two large-scale vortices. 

The absolute value of the vertical vorticity for large-scale vortices reaches ~0.033 s^−1^, and the characteristic dimensions of the vortex L~6 cm. From this data one can estimate that the Reynolds number Re~7000. So, the large-scale vortices are characterized by a high Re that indicates to the strong nonlinear interaction between the vortices. Moreover, one should wait that when the energy pumping from the bulk was switched off, at temperatures T far above 2.2 K, the mutual interaction between vortices and their interaction with the vessel bottom and sidewalls should lead to the power-law dependence of the energy decay on time [17,18,19].

**Figure 7:**
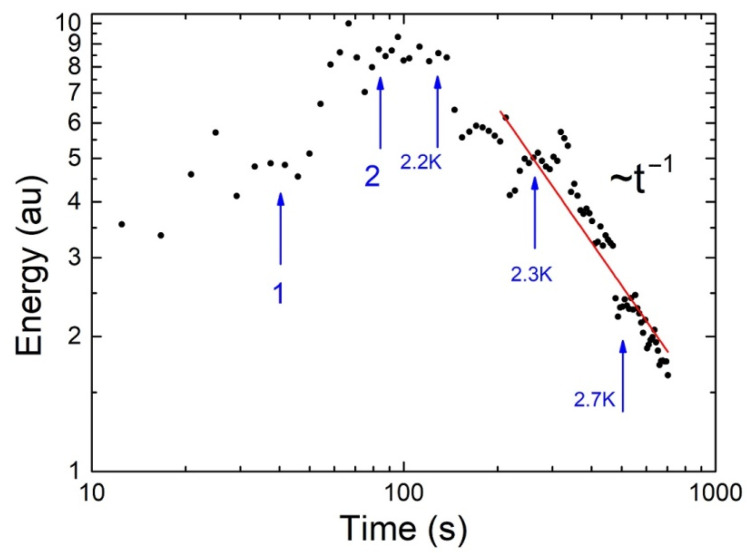
Time dependence of the total energy of the vortex system on the surface of the liquid. Energy E is presented in arbitrary units.

The time dependence of the total energy of the vortex motion on the liquid surface E(t) observed in this experiment is shown in Figure 7. The moment of the crossing T_λ_ and transition from the superfluid to the normal state is taken as a reference point. The energy E is shown in arbitrary units. Each energy point was calculated at a certain point in time over the entire velocity field obtained by averaging the movement of tracers for 4.2 s in the time interval up to ~140 s, and then by averaging for 8.4 s in the time interval after 140 s.

In Figure 7, arrows 1 and 2 mark the times corresponding to the times in Figure 5 and Figure 6, respectively. After ~150 s, the decay of the energy of the vortex motion on the surface could be represented by the power law E~(1/t)^n^ with the exponent close to n ≈ 1, as expected [18,19]. The blue arrows in Figure 7 show the temperature of liquid layer near the vessel bottom T_b_ at the corresponding time points.

## 4. Conclusions and Outlook

The described experimental approach for studying the vortex flow on the surface generated by the buoyancy-driven non-Boussinesq convection in the bulk of a normal liquid helium has several principal advantages. Superfluid helium provides uniform initial conditions throughout the entire bulk of the liquid at the beginning of the experiment. Next, on heating the liquid layer in the vessel above T_λ_, the energy is pumped into a vortex system in a natural way due to development of the thermogravitational RBC in He-I. The pumping stops working when the temperature of the liquid at the vessel bottom T_b_ reaches the temperature at which the maximum density of the liquid is realized. The free surface of liquid helium enables studying the evolution of vortices on the He-I surface over a long period of time.

The direct measurements have shown that the emergence of the RBC in the heated from above layer of normal helium at temperatures just above T_λ_ is accompanied by the appearance of a vortex flow on the free liquid surface. Nonlinear interaction between vortices on the surface and in the volume during the onset of turbulent convection in the layer (i.e., in the presence of energy transfer from the volume to the system of surface vortices) leads to the formation of two large-scale vortices (vortex dipole) on the liquid surface. 

Any turbulent flow in the volume of He-I layer heated from above decays rapidly with an increase in the layer temperature above T_m_. Note that in the absence of bulk pumping, at Tb > Tm, the total energy of large-scale vortices with characteristic dimensions twice the layer thickness (in the 2D situation according to the works in [17,18,19]) decreased with time as E~(1/t)^n^, where the exponent n varied from 1 to 2 in different experiments. 

The power-law damping of energy could be explained by the nonlinear interaction between large-scale vortices and their interaction with the bottom and sidewalls of the vessel, as well as by the formation of small-scale vortices on the liquid surface.

Preliminary results of the measurements were presented in part at the conference on heat and mass transfer [20], and some of them were included in the collection of articles in the special issue of JLTP dedicated to the 90th anniversary of professors D. Lee and J. Reppy.

## Data Availability

Data are contained within the article.

## References

[1] Landau L.D., Lifshitz E.M. (1987). Course of Theoretical Physics, Fluid Mechanics.

[2] Walden R.W., Ahlers G. (1981). Non-Boussinesq and penetrative convection in a cylindrical cell. J. Fluid Mech..

[3] Ahlers G., Grossmann S., Lohse D. (2009). Heat transfer and large scale dynamics in turbulent Rayleigh-Benard convection. Rev. Mod. Phys..

[4] Roche P.-E. (2020). The ultimate state of convection: A unifying picture of very high Rayleigh numbers experiments. New J. Phys..

[5] Moller S., Resagk C., Cierpka C. (2021). Long-time experimental investigation of turbulent superstructures in Rayleigh–Bénard convection by non-invasive simultaneous measurements of temperature and velocity fields. Exp. Fluids.

[6] Chilla F., Schumacher J. (2012). New perspectives in turbulent rayleigh-benard convection. Eur. Phys. J. E.

[7] Niemela J.J., Sreenivasan K.R. (2006). The use of cryogenic helium for classical turbulence: Promises and hurdles. J. Low Temp. Phys..

[8] Pel’menev A.A., Levchenko A.A., Mezhov-Deglin L.P. (2019). Vortices on the surface of normal he i generated by the rayleigh–bénard thermogravitational convection in the bulk of a liquid. JETP Lett..

[9] Pelmenev A.A., Levchenko A.A., Mezhov-Deglin L.P. (2020). The evolution of vortices on the surface of normal He I. Low Temp. Phys..

[10] Donnelly R.J., Barenghi C.F. (1998). The Observed Properties of Liquid Helium at the Saturated Vapor Pressure. J. Phys. Chem. Ref. Data.

[11] Peshkov V.P., Borovikov A.P. (1966). Measurement of the λ-transition temperature and density maximum of liquidHe-4. Sov. Phys. JETP.

[12] Mezhov-Deglin L.P., Trickey S.B., Adams E.D., Dufty J.W. (1977). Kapitza Resistance at a Solid Helium-Copper Interface under Heavy Thermal Loads. Quantum Fluids and Solids.

[13] Baudouy B., Four A. (2014). Low temperature thermal conductivity of aluminium alloy 5056. Cryogenics.

[14] Kuznetsov E.A., Spektor M.D. (1980). Weakly supercritical convection. J. Appl. Mech. Tech. Phys..

[15] Levchenko A.A., Lebedeva E.V., Mezhov-Deglin L.P., Pelmenev A.A. (2019). Self-organization of neutral particles on the surface of superfluid He-II. Low Temp. Phys..

[16] Filatov S.V., Levchenko A.A., Brazhnikov M.Y., Mezhov-Deglin L.P. (2018). A Technique for Registering Wave and Vortex Motions on a Liquid Surface. Instrum. Exp. Tech..

[17] Falkovich G., Boffetta G., Shats M.A., Lanotte A.S. (2017). Introduction to Focus Issue: Two-Dimensional Turbulence. Phys. Fluids.

[18] Filatov S., Levchenko A., Likhter A., Mezhov-Deglin L. (2019). Quasi-adiabatic decay of vortex motion on the water surface. Mater. Lett..

[19] Filatov S.V., Levchenko A.A., Mezhov-Deglin L.P. (2020). Formation and decay of Vortex Motion on a Liquid Surface. JETP Lett..

[20] Pelmenev A.A., Levchenko A.A., Mezhov-Deglin L.P. The Rayleigh-Benard convection in the bulk and vortex flow on the surface of a normal liquid helium layer. Proceedings of the 15th International Conference on Heat Transfer, Fluid Mechanics and Thermodynamics.

[21] Demuren A., Grotjans H. (2009). Buoyancy-Driven Flows-Beyond the Boussinesq Approximation, Numerical Heat Transfer, Part B: Fundamentals. Int. J. Comput. Methodol..

[22] Paolitto G., Greco C.S., Astartita T., Cardone G. (2021). Experimental determination of the 3-D characteristic modes of turbulent Rayleigh-Benard convection in a cylinder. J. Fluid Mech..

